# Supplementary material for: From sequence to enzyme mechanism using multi-label machine learning
Source: BMC Bioinformatics. 2014 May 19;15:150. doi: 10.1186/1471-2105-15-150 (PMC4229970; doi:10.1186/1471-2105-15-150)
Supplement: Additional file 2 — Java code of ml2db. Additional file ml2db_code.tar.gz contains the Java source code to run the multi-label machine learning experiments and save the results to database. The code’s Javadoc is included. [file 1471-2105-15-150-S2.zip › additional file 2/ml2db/ml2db/doc/test/mulan/predict/MulanPredictTest.html]

MulanPredictTest


---


|  |  |  |
| --- | --- | --- |
| **Overview** \| **Package** \| **Class** \| **Use** \| **Tree** \| **Deprecated** \| **Index** \| **Help** | |  |
| **PREV CLASS**   NEXT CLASS | **FRAMES**    **NO FRAMES**     **All Classes** |  |
| SUMMARY: NESTED \| FIELD \| CONSTR \| METHOD | DETAIL: FIELD \| CONSTR \| METHOD |  |


---


## test.mulan.predict Class MulanPredictTest

```
java.lang.Object
  junit.framework.Assert
      junit.framework.TestCase
          test.mulan.predict.MulanPredictTest
```

**All Implemented Interfaces:**: junit.framework.Test

---

``` public class MulanPredictTest extends junit.framework.TestCase ```

Train arff: inst1, inst2 etc. att 1, 3, 4, 5 class 1.1.1.1, 2.2.2.2
Test arff: instA, instB, instC etc. att 2, 3 class 2.2.2.2, 3.3.3.3
Test data filtered by train: cannot contain att 2, must contain att1, 3, 4, 5
Types comment: change at eclipse > java > code style > code template > Types

---

**Constructor Summary**

`MulanPredictTest()`


| **Method Summary** | |
| --- | --- |
| `static MulanPredict` | `getMulanPredict(int trainId, int testId)` |
| `static MulanPredict` | `getMulanPredictFromArffId()` |
| `static MulanPredict` | `getMulanPredictFromInstancesBRkNN()` |
| `static MulanPredict` | `getMulanPredictFromInstancesBRkNNAL()` |
| `static MulanPredict` | `getMulanPredictOnAttributesFilteredFiles()` |
| `static MulanPredict` | `getPredictor(MulanLearner learner)` |
| `void` | `testHeader(java.lang.String predictions)` |
| `void` | `testPrintPredictionsAttributesFiltered()` |
| `void` | `testPrintPredictionsConstructor1()` |
| `void` | `testPrintPredictionsConstructor2()` |

**Methods inherited from class junit.framework.TestCase**

`countTestCases, getName, run, run, runBare, setName, toString`

**Methods inherited from class junit.framework.Assert**

`assertEquals, assertEquals, assertEquals, assertEquals, assertEquals, assertEquals, assertEquals, assertEquals, assertEquals, assertEquals, assertEquals, assertEquals, assertEquals, assertEquals, assertEquals, assertEquals, assertEquals, assertEquals, assertEquals, assertEquals, assertFalse, assertFalse, assertNotNull, assertNotNull, assertNotSame, assertNotSame, assertNull, assertNull, assertSame, assertSame, assertTrue, assertTrue, fail, fail, failNotEquals, failNotSame, failSame, format`

**Methods inherited from class java.lang.Object**

`equals, getClass, hashCode, notify, notifyAll, wait, wait, wait`

**Constructor Detail**

### MulanPredictTest

```
public MulanPredictTest()
```


**Method Detail**

### testHeader

```
public void testHeader(java.lang.String predictions)
```

---


### testPrintPredictionsAttributesFiltered

```
public void testPrintPredictionsAttributesFiltered()
```

---


### testPrintPredictionsConstructor1

```
public void testPrintPredictionsConstructor1()
```

---


### testPrintPredictionsConstructor2

```
public void testPrintPredictionsConstructor2()
```

---


### getMulanPredict

```
public static MulanPredict getMulanPredict(int trainId,
                                           int testId)
```

---


### getMulanPredictFromArffId

```
public static MulanPredict getMulanPredictFromArffId()
```

---


### getMulanPredictFromInstancesBRkNN

```
public static MulanPredict getMulanPredictFromInstancesBRkNN()
```

---


### getMulanPredictFromInstancesBRkNNAL

```
public static MulanPredict getMulanPredictFromInstancesBRkNNAL()
```

---


### getMulanPredictOnAttributesFilteredFiles

```
public static MulanPredict getMulanPredictOnAttributesFilteredFiles()
```

---


### getPredictor

```
public static MulanPredict getPredictor(MulanLearner learner)
```


---


|  |  |  |
| --- | --- | --- |
| **Overview** \| **Package** \| **Class** \| **Use** \| **Tree** \| **Deprecated** \| **Index** \| **Help** | |  |
| **PREV CLASS**   NEXT CLASS | **FRAMES**    **NO FRAMES**     **All Classes** |  |
| SUMMARY: NESTED \| FIELD \| CONSTR \| METHOD | DETAIL: FIELD \| CONSTR \| METHOD |  |


---
